# Focus on pain in the blind

**DOI:** 10.1016/j.pain.2013.06.029

**Published:** 2013-10

**Authors:** Flavia Mancini

**Affiliations:** Institute of Cognitive Neuroscience, University College London, 17 Queen Square, London WC1N 3AR, UK

Until recently, the cross-modal consequences of unisensory deprivation have been extensively studied in almost every sensory domain other than nociception. In this issue of PAIN®, Slimani et al. [8] explore, for the first time, the sensory consequences of congenital blindness on thermal sensitivity and pain perception.

Their study has provided evidence that congenitally blind participants are hypersensitive to pain. Slimani et al. [8] observed lower pain thresholds to cold and heat in blind participants, relative to matched sighted volunteers, as well as higher ratings of pain intensity in response to suprathreshold laser stimuli. Interestingly, detection thresholds of innocuous warmth and cold were not different between blind and sighted participants. This suggests that thermal hypersensitivity in blindness could be specific to pain. These results were replicated in 2 European populations, Italian and Danish, which were previously reported to be differently sensitive to pain [7].

Slimani et al. [8] also assessed attitudes and responses towards signals of threat in daily life in both blind and sighted participants. The results from 2 questionnaires (Pain Anxiety Symptoms Scale, and Pain Vigilance Awareness Questionnaire) show that blind participants were more attentive to potential threat in daily life than their sighted counterparts.

The novel finding of pain hypersensitivity in blindness has several important implications for both basic and clinical science. This study is noteworthy for research on multisensory interactions and plasticity because it shows a strong link between vision and pain. This link is supported by a previous report of increased pain sensitivity in sighted volunteers who were temporarily visually deprived [11]. Studies conducted on sighted participants also showed that the visual context can modulate the perception both of acute [3] and chronic pain [4]. The next step is to understand the nature of the interaction between visual loss and pain sensitivity. Which aspect of pain processing is involved in the interplay with vision, and what is its neural basis?

Fig. 1 shows 2 putative mechanisms that could underlie the hypersensitivity to pain reported by Slimani et al. [8]. First, pain hypersensitivity could reflect cross-modal plasticity of brain circuitry after blindness. Indeed, visual loss from birth induces structural and functional changes in brain connectivity [2], [6], which may underlie the increased tactile sensitivity previously reported in blindness [2]. Future studies could investigate whether similar mechanisms of brain reorganization underlie changes in tactile and nociceptive sensitivity in blind people. Enhanced attention to threat and anxiety experienced by blind participants in daily life would then be the consequence of the hypersensitivity to sensory stimuli (Fig. 1a). Alternatively, the hypersensitivity to pain in blind individuals might be caused, in first instance, by uncertainty about threat, due to lack of vision (Fig. 1b). Uncertain expectation of pain is often associated to anxiety and increased pain sensitivity [5]. The hypersensitivity to pain would thus actually reflect a hypersensitivity to threat rather than processes specific to Aδ pathways. This hypothesis would predict that top-down, descending modulation from anterior cortical regions [5], [9] would amplify incoming sensory signals (Fig. 1b). Finally, a combination of bottom-up and top-down processes might underlie the results described by Slimani et al. [8].

**Fig. 1:**
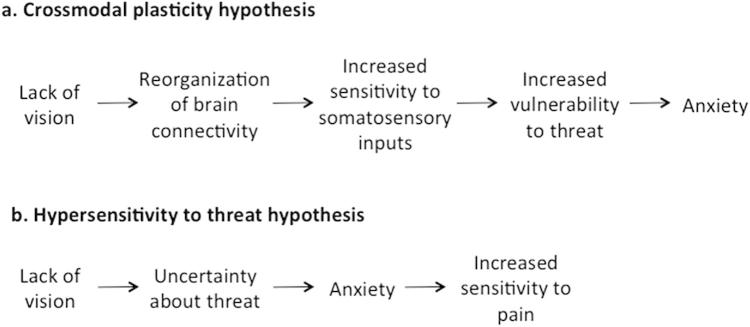
Putative mechanisms underlying pain hypersensitivity in blindness.

The ‘hypersensitivity to threat’ hypothesis (Fig. 1b) predicts that the increased sensitivity to potentially harmful stimuli would not be specific to the Aδ pathway, and so could theoretically affect every sensory stimulus presented in a context of danger. However, the authors found no significant differences between the detection thresholds of innocuous thermal stimulation in sighted and blind participants. Importantly, innocuous and noxious thresholds were tested in separate blocks, cued for the level of stimulation. Hence, it cannot be excluded that the lack of modulation of innocuous thresholds is a consequence of knowing in advance that one stimulus would not be harmful. Future studies using event-related designs, in which all the stimuli would be randomized and unpredictable, could shed light on the role of pain expectation on thermal perception in blindness. Specifically, the ‘hypersensitivity to threat’ hypothesis would predict increased sensitivity to innocuous stimulation in blind participants, when the stimuli are unpredictable.

The study by Slimani et al. [8] also deserves attention for its clinical implications. The World Health Organization estimates that in 2012, 39 million of people were blind [10]. If the results by Slimani et al. [8] are confirmed in larger samples of blind individuals, it becomes of primary importance to examine the potential short- and long-term risks of being hypersensitive to pain due to blindness. Could it increase the risk of developing both acute and chronic pain conditions?

Unfortunately, the assessment of thermal sensitivity requires expensive equipment, which could thwart routine testing of large samples of people. However, there are several low-cost alternative methods (e.g., punctate probes to stimulate Aδ mechanical fibers [1]) that could be used to routinely assess pain perception outside pain laboratories and clinical settings. These methods are often easy and quick to use, but they do not provide selective-stimulation Aδ fibers. Therefore, similar applied studies can complement but not substitute rigorous laboratory testing using nociceptive selective stimulation.

The hope is that the work by Slimani et al. [8] will open the door to pain investigations into the world of sensory loss, left unexplored for too long.

## Conflict of interest statement

The author is aware of no conflicts of interest regarding this commentary.
